# Social determinants of under-5 child health: A qualitative study in Wolkayit Woreda, Tigray Region, Ethiopia

**DOI:** 10.1371/journal.pone.0218101

**Published:** 2019-06-13

**Authors:** Atakelti Abraha, Anna Myléus, Peter Byass, Asmelash Kahsay, John Kinsman

**Affiliations:** 1 Tigray Health Bureau, Tigray and Ethiopian Health Insurance Agency, Addis Ababa, Ethiopia; 2 Umeå Centre for Global Health Research, Department of Public Health and Clinical Medicine, Umeå University, Umeå, Sweden; 3 Family Medicine, Department of Public Health and Clinical Medicine, Umeå University, Umeå, Sweden; 4 Institutes of Applied Health Sciences, School of Medicine and Dentistry, University of Aberdeen, Aberdeen, United Kingdom; 5 MRC/Wits Rural Public Health and Health Transitions Research Unit, School of Public Health, Faculty of Health Sciences, University of the Witwatersrand, Johannesburg, South Africa; 6 Tigray Regional Health Bureau, Tigray, Ethiopia; 7 Department of Public Health Sciences, Global Health (IHCAR), Karolinska Institute, Stockholm, Sweden; Makerere University, UGANDA

## Abstract

Despite the significant reductions seen in under-5 child mortality in Ethiopia over the last two decades, more than 10,000 children still die each year in Tigray Region alone, of whom 75% die from preventable diseases. Using an equity lens, this study aimed to investigate the social determinants of child health in one particularly vulnerable district as a means of informing the health policy decision-making process. An exploratory qualitative study design was adopted, combining focus group discussions and qualitative interviews. Seven Focus Group Discussions with mothers of young children, and 21 qualitative interviews with health workers were conducted in Wolkayit district in May-June 2015. Data were subjected to thematic analysis. Mothers’ knowledge regarding the major causes of child mortality appeared to be good, and they also knew about and trusted the available child health interventions. However, utilization and practice of these interventions was limited by a range of issues, including cultural factors, financial shortages, limited female autonomy on financial resources, seasonal mobility, and inaccessible or unaffordable health services. Our findings pointed to the importance of a multi-sectoral strategy to improve child health equity and reduce under-5 mortality in Wolkayit. Recommendations include further decentralizing child health services to local-level Health Posts, and increasing the number of Health Facilities based on local topography and living conditions.

## Introduction

The Millennium Development Goals (MDGs) were instrumental in increasing investment and action for child survival. Globally, the under-5 child mortality has decreased from an estimated 91 deaths per 1000 live births in 1990 to 43 per 1000 live births in 2015[1]. However, wide differences in the reduction of mortality and utilization of most maternal and child health indicators still exist within and across countries; these are often linked to underlying factors such as cultural and socio-economic characteristics. Such differences in health outcomes—which can also be seen as health inequities—are socially produced, systematic in their distribution across the population, and unfair [2]. On this basis, the World Health Organization (WHO) considers efforts to address inequities as key components of global efforts aimed at improving maternal, new-born and child health and survival rates [3]. Bringing an end to preventable deaths of new-borns and under- 5 year children is also a priority in the Sustainable Development Goals [4].

Income differences within countries and between households are key determinants of inequities in child health. Better economic status reduces the chances of child sickness by improving a child’s nutritional status, uptake of child health interventions, and health-seeking behaviour [5–6], as well as increasing parental capacity and willingness to pay for better care [7–8]

The decision-making power of women, in relation to the use of household resources and time spent seeking medical care, serves as an important determinant for child health. In countries where women are economically dependent on their husband, utilization of maternal and child health services is lower, resulting in poorer new-born and child health outcomes [9]. Studies in Ethiopia and Gambia indicate that child mortality may be between three and twelve times higher respectively, among mothers with limited decision-making power[10–11]

Access to health services such as availability of services, geographic accessibility, travel time, as well as affordability, determine child health inequity. These factors mostly favour those who live closer to health facilities, and those who can afford both the direct and indirect costs of the service as well as the opportunity costs associated with receiving the service [12–14]

Knowledge about the causes of illness and their preventive strategies, as well as trust in services can determine usage rates of health care and there by affect child health status. Lack of knowledge, perceived poor quality health care, and inadequate education to the parents [15] can lead to misperceptions regarding illness and the effectiveness of care, loss of trust in the health care system, and these in turn can lead to use of potentially under-trained traditional healers [16–17].

Some of the key structural and intermediate determinants that have a bearing on inequities of under-5 child health in parts of Ethiopia include cultural and religious practices. These have been identified as contributors to high rates of maternal, neonatal and child mortality, through their acting as barriers to utilization of maternal and child health interventions because of a preference for home delivery over facility delivery, and of traditional healers over biomedical treatment [5].

### Policy context

The Government of Ethiopia has long worked to reduce economic disparities in the population through economic reforms and efforts to re-orient the health services to reach the rural and the poorer populations in health promotion, disease prevention, and curative services. These efforts have included new initiatives such as the Health Extension Program (HEP) and health care financing strategies, among others.

**Fig 1:**
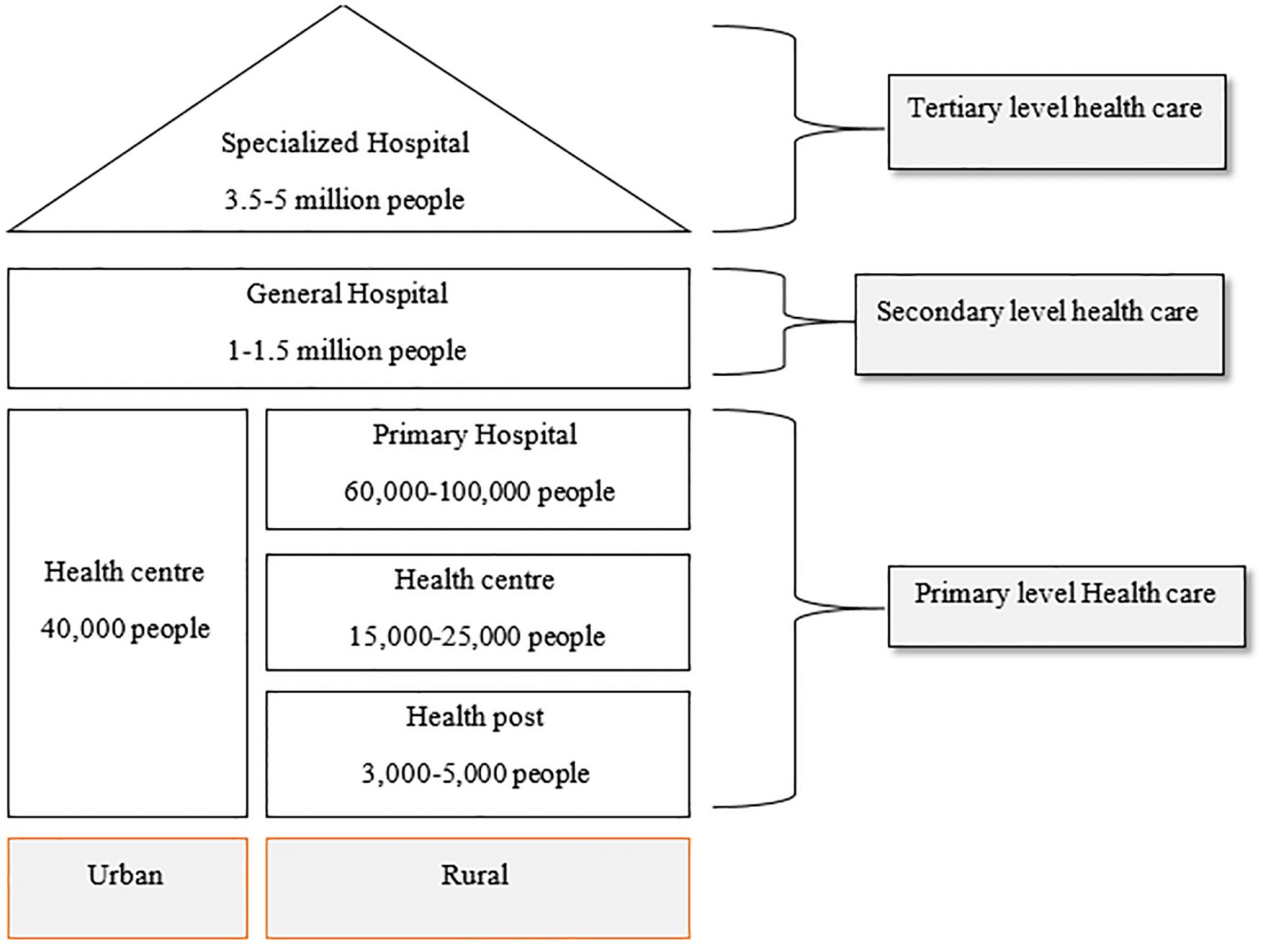
Three-tier Ethiopian health care system (Source: Ethiopian health sector development plan 2010–2015).

The current Ethiopian health system is decentralized, with three tiers “Fig 1”. These include, at the primary care level, a Woreda-based (District-based) system that includes one Primary Hospital, Health Centres, and Health Posts form the primary health care. The secondary care level includes General Hospitals reached through referral, while the tertiary level provides care at teaching and Specialised Hospitals. In 2015, there was a total of 16,440 Health Posts, 3,547 Health Centers and 311 Hospitals in the country, providing access to health care for more than 95 percent of the population [18].

The HEP, initiated in 2005, is an innovative approach designed to address health care inequities in remote and rural communities, ethnic minority groups, and poor semi-urban residents [19]. The program focuses on women and children and aims to enable communities to promote their own health through health education, community participation; and provision of seventeen high impact health promotion, disease prevention and curative services.

Two women who have completed Grade 10 in school are recruited locally and trained for one year—called Health extension Workers (HEWs)—provide the HEP for the population of one Kebele/sub-district (= 3,000–5,000 people) [20]. To improve the implementation capacity of the health sector by engaging communities in the identification of challenges and corresponding strategies to address them; the HEWs organize around 30 women each who are residing in the same neighbourhood within what is called the Health Development Army (HDA). The HEWs support them, identify their household and group health needs, plan their health interventions, and lead their weekly meetings.

All HEP services are provided free of charge, and there is an exemption of charges for selected public health importance diseases. A waiver system also exists for those who are unable to pay are implemented at all higher level public health facilities [21]. Additionally, since 2012, there has been a Community Based Health Insurance system in the country that requires members to contribute 9 USD per year to cover most health services for their family, while those who are unable to pay are covered by the local and regional Government. This ensures sustainable financial accessibility of services to the poor.

These and other initiatives have collectively led to improvements in the utilization of maternal and child health services in Ethiopia. Under-5 child mortality in the country has been reduced to 67 deaths per 1000 live births in 2015 [22], down from 204 deaths per 1,000 live births in 1990. Despite the gains made, however, still more than 200,000 under-5 year children in Ethiopia—of whom more than 10,000 are in Tigray region—still die every year and inequities in mortality rates across socio-demographic groups and geographical areas remain challenges. Children born to under-educated women and those living in the lowest economic quintiles mothers die at 2.6 times the rate of their higher educated and wealthier counterparts [23]. Within Tigray region, routine reports indicate lower utilization of maternal and child health services and latrine coverage in Wolkayit Woreda; and higher malnutrition and neonatal death rates than the regional average [24].

**Fig 2:**
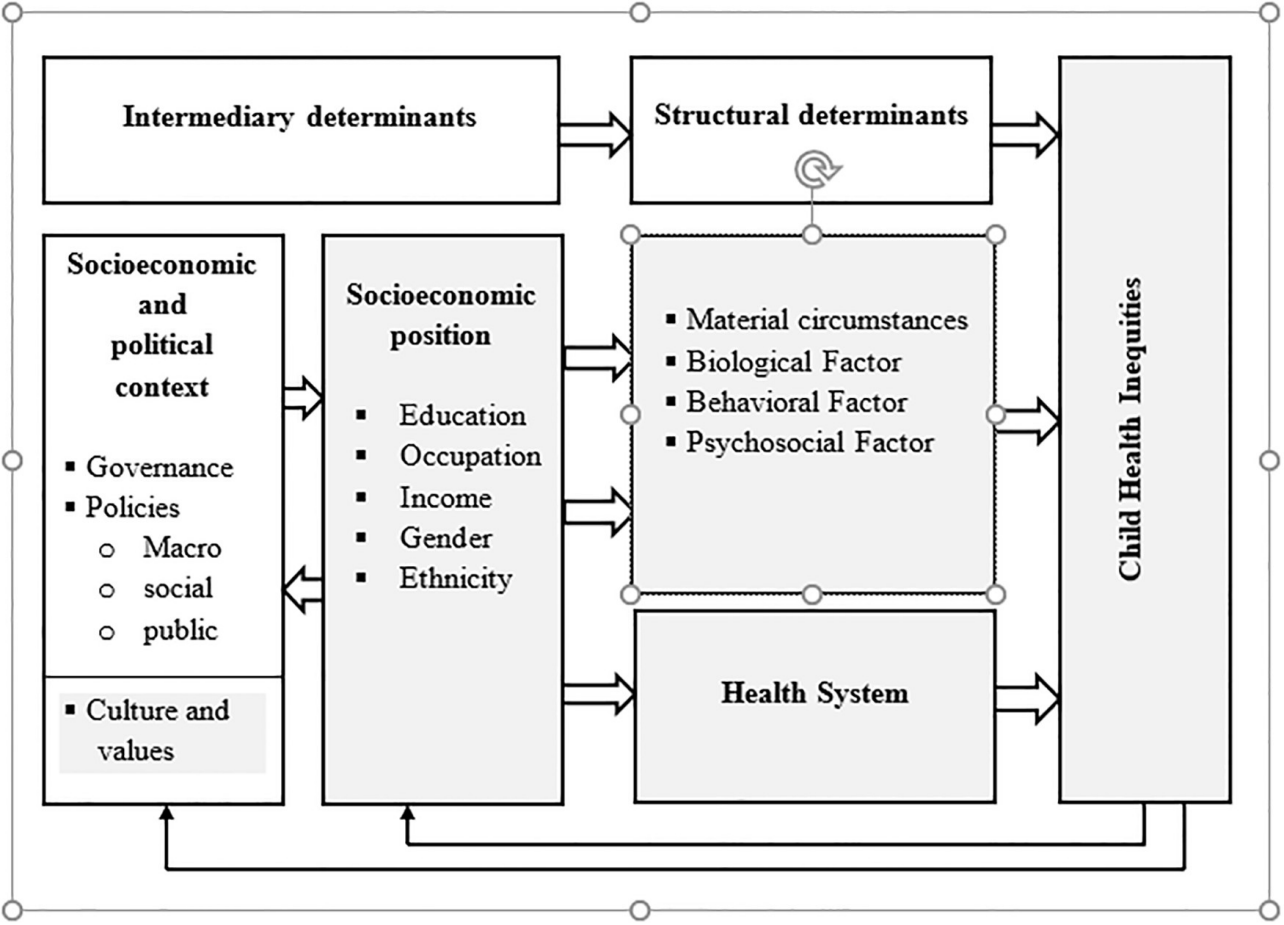
Conceptual framework of social determinants for child health inequities (adopted from Solar and Irwin, 2010).

This is the background for the present, exploratory qualitative study, which aims to investigate the social determinants of child health in Wolkayit Woreda. As explained above, the survival, health and well-being of children is profoundly affected by their parent’s socio-economic status. Therefore, we adopt the conceptual framework for social determinants of health inequities formulated by Solar and Irwin [25] as the theoretical basis for our study “Fig 2”.

The framework illustrates the causal relationship of macro-level structural determinants such as the governance and policies with the intermediary determinants within the health system; and more proximal factors such as household material circumstances. All of these determinants play key roles in determining child health. This study focuses on the determinants from the community and the health system perspectives as indicated in the framework.

## Methods and materials

### Ethics approval and consent to participate

Ethical approval and a permission letter were received from the Institutional Review Board (IRB) of Mekelle College of Medical Sciences Ref no ERC 0645/2015. Permission was sought from Tigray Regional Health Bureau and Wolkayit Woreda health office. With approval of the IRB in the procedure, verbal informed consent was obtained because the FGD participants were illiterate and preference of the qualitative interviewees. The consent read to the participant and responses of participants were tape recorded. All individual identifiers were removed during transcription to ensure anonymity.

### Study setting and design

In Ethiopia, the Woreda (District) is the third-level administrative division of Government. Woredas are further subdivided into a number of wards or neighbourhood associations called Kebeles, which comprise the smallest unit of local Government. Based on the 2007 census conducted in Ethiopia, Wolkayit Woreda in western Tigray Region has a total population of 140,000, of whom 49.2% were women, 92.3% were rural inhabitants, 97.3% were orthodox Christian and 2.7% were Muslim [26].

With an area of 3,375 km^2^, Wolkayit Woreda has a population density of 41/km^2^. Wolkayit Woreda is known for its mountains and cliffs. Only 33% of the outlying Kebele can be reached by paved roads, and telecommunication coverage reaches just 50% of the population [27]. There are 26 Health Posts, eight Health Centres and one Primary Hospital in the Woreda, but people requiring advanced care must travel to a General Hospital more than 100 km from the Woreda capital.

This is an exploratory qualitative study that combined Focus Group Discussions (FGDs) and one-to-one key informant qualitative interviews, conducted from May-June 2015 in Wolkayit Woreda. Using the Woreda Health office profile, five kebeles were purposively selected, each with a functional Health Post: Two were within 10 km of the Woreda capital (where the primary hospital and highest level health services are available), and the other three were over 10 km from the Woreda capital. This approach allowed us to examine differences related to the remoteness of a population.

### Sampling and study population

Women who lived in the kebele and had given birth in the past five years, and who were living within five kilometres of their Kebele health facility or health post were eligible to participate in the FGDs. They were identified on a purposive basis with the help of the health extension workers, and they were contacted a few days before the planned FGD to explain the objective of the study and request their participation. For the one-to-one qualitative interviews, managers and health workers of different discipline at primary and higher level of health facilities were contacted by the principal investigator two weeks before the interview. The most senior health worker from eligible health facilities was invited to participate.

### Data collection and analysis

Semi-structured guiding questions were prepared for the FGDs and qualitative interviews in English, and then translated into the local language (Tigrinya) S1 Table. These were pre-tested in a non-study area, and problems relating to, for example, the sequence of questions, conceptually similar questions, and sensitive wording were corrected S2 Table. The COREQ guidelines [28] were used throughout the research process to ensure consistency and quality. Three of the authors (AA, JK, and AK)—two of whom are from Tigray region themselves and are thus familiar with the culture and fluent in local language—conducted the FGDs and qualitative interviews face-to-face. The principal Investigator (AA) and AK have conducted many FGDs and interviews; AA recently completed course on qualitative research methods; and (JK) is an experienced qualitative researcher who has authored a number of qualitative studies.

FGDs were conducted in community halls, and qualitative interviews were conducted at the health facility where the interviewee worked. Non-participants were politely asked to give privacy in some facilities where two or more health workers share a room. Discussions were digitally recorded and complementary notes were taken to record observations about the participants’ comments and their interactions.

The principal investigator and the moderator transcribed each interview and FGD word-for-word in the local language, Tigrinya, and then translated the transcripts into English. The translations were verified by listening to the recordings while re-reading the transcripts. Thematic analysis [29]was used to analyse the data. The data were coded manually following the set of *a priori* themes laid out in the questions, such as preference of place of delivery, common childhood illnesses, and mother’s actions for their sick child.

Data from the FGDs and qualitative interviews were analysed separately. Four main themes representing the material from the FGD participants and qualitative interviews are presented in the Findings section, with illustrative quotes included to support the main findings. The themes are based on four of the major components of the childhood health inequities conceptual framework, as presented in Fig 1.

## Findings

### Participant characteristics

A total of seven FGDs was held, after which the research team agreed that we had reached saturation. Out of the 88 women contacted, 86 (97.7%) agreed to participate in one of the seven FGDs (four including women from <10km of the Woreda centre, and three from >10km). The median age of the FGD participants was 35 years. None of the FGD participants had formal education since this was introduced only 25 years ago to this remote area.

One-to-one qualitative interviews were conducted with managers and health workers of different categories from five Health Posts, three Health Centres, one Primary Hospital, one General Hospital that serves the Woreda. Twenty-one health workers were contacted and all participated in the interview “Table 1”. Seven out of ten of the HEWs and seven out of eleven of the other health workers had less than one year’s work experience, reflecting very high levels of staff turnover in this Woreda.

**Table 1:** Qualitative interview participants, by distance to the Woreda centre and profession.

Profession	Within 10 Km of the Woreda centre		More than 10 Km from Woreda centre		Total	
	M	F	M	F	M	F
Health extension workers	0	4	0	6	0	10
Clinical nurses	1	0	0	2	1	2
Midwifes	0	1	0	2	0	3
Clinicians	0	0	2	0	2	0
Health managers	2	0	1	0	3	0
Total	3	6	3	9	6	15

On average, the FGDs took 46 minutes (39–67 minutes), while the qualitative interviews took 34 minutes (19 to 47 minutes). Even though participants were initially stratified by distance from the Woreda centre, we subsequently combined the results of both age groups and distance groups, as no notable differences between the groups were identified during analysis.

Major emergent themes of the findings of the study are summarized in “Table 2” under the four major (and in some respects, overlapping) themes derived from the conceptual framework. They are then presented in more detail below, alongside their associated sub-themes.

**Table 2:** Determinants of under-5 child health in Wolkayit Woreda under four themes emerged out of the FGD and qualitative interview.

Themes	Sub-themes
Cultural factors	Family members discourage women to be seen naked Restriction of movement out of home after delivery Withholding of information regarding neonatal death Limited role of husband in child care
Financial factors	Poor household economy Low female autonomy
Behavioural factors	Seasonal mobility of members of household Single motherhood
Health system factors	Inaccessible Health Centres and Hospital Unavailability of services Unaffordable cost of services

### Theme: Cultural factors

Our study identified a set of cultural factors that undermine child health. These factors were not openly discussed by the FGD participants; and HEWs were also reluctant to discuss them. Therefore, these findings are derived from the interviews with the more senior and qualified health workers, nearly all of whom emphasized cultural issues as being major determinants for low utilization of health care opportunities.

#### Family members discourage women to be seen naked

Although all categories of respondents confirmed that the acceptance and utilization of facility delivery has increased over time, home delivery is still perceived as favourable, in part because family members do not want women to be seen naked by strangers (especially male health workers) during labour. Clearly, there are significant risks inherent in home deliveries for both mother and child. A Primary health care unit director explained, “Most births occur at home, partly because family members encourage the mother to deliver at home [so as] not to be seen naked at the health facility; and most neonatal deaths occur at home.”

#### Restriction of movement outside the home after delivery

Avoiding any movement out of home for at least 40 days after delivery to prevent the mother and her new-born being exposed to the wind and sun is still a common practice by a majority of mothers. Health workers pointed to this restriction of movement as a major challenge for post-natal follow-up. A midwife said that, “Some mothers are reached during this period by health workers during home visits, but the majority, especially those who deliver at home, do not receive post-natal care; and sick neonates are rarely brought to a health centre for treatment.” A clinical nurse explained, “It is not possible to provide the necessary care for the baby and mother who deliver at home because the mother is restricted at home for 40 days after delivery.” Another nurse reported, “Many women do not come for post-natal care; they tell me they do not want to be exposed while the baby is healthy.”

#### Withholding of information regarding neonatal death

The FGD participants displayed good knowledge about the causes of childhood morbidity and mortality, and associated preventive strategies. They know most of the diseases that our study identified for discussion by local names. For example, we heard of *“Ankelish”* for measles, *“Aytikreb”* for whooping cough, “*Aso*” for malaria, *“Nifas”* for pneumonia *“Wetetie”* for chickenpox, and “*Wutsiat*” for diarrhoea. However, the FGD participants and HEWs were hesitant to talk about child deaths; and many withdrew from the discussions regarding neonatal death. Those who were comfortable addressing the topic reported that they felt that the problem was no longer very prevalent these days. As one HEW explained, “Eighty-eight mothers delivered in the past eight months, and all mothers and neonates are in a good health…. I do not agree that we have high child mortality.” An FGD participant added that, “Only one, a two-year old child died this year, even though we had outbreak of ‘Ankelish [measles]… Otherwise child death is rare now days in our catchment.” However, more senior health workers suggested that the under-5 mortality is higher in the Woreda compared to other Woredas, due to higher home delivery rates and repeated measles outbreaks. But child deaths are not openly discussed in the community, and they are often not reported. This attitude contributes to underestimates of neonatal and child mortality, which in turn hinders the development of appropriate policies.

A Primary health care unit director explained, “there were 12 deaths in the health centre alone in one year …. only six home deaths were reported by HEWs in 15 kebele. I can imagine many more unreported deaths in the nine kebele where there are no HEWs.” A clinician added, “Most of neonatal and child deaths occur at home, and these events are not openly discussed; partially not to aggravate mothers’ pain at losing her baby, but also for fear of stigma related to communities’ perception that the death of a new born may be due to evil spirits; therefore, they are not reported to the health system…. the deaths are under-estimated.”

#### Limited role of husband in child care

Culturally, the role of husbands is limited to providing food and other basic necessities for the household. All the responsibilities regarding the health of the family, child feeding, sanitation, and seeking medical care fall to the mother, with little or no support from the men. Lower male health literacy as compared to that of women (which is enhanced by the HEWs, and which focuses primarily on women’s behavioural change) contributes to the problem. Most FGD participants agree that men should not participate in child care. As one said, “It is not customary for men to care children, there are few …. but mostly feeding, clothing and schooling is the responsibility of the mother.”

### Theme: Financial factors

Women often face financial shortages due to limited employment opportunities, single motherhood, and a lack of autonomy to make decisions about household resources.

From the FGD participant discussions, it was clear that interventions that incur costs to the household like improved child feeding practices and latrine construction are poorly implemented; whereas, interventions that are available without cost, such as immunizations, malaria medications and HIV prevention services have better uptake. FGD participants reported that the solution to increase the uptake of services would be for the Government to supply food resources and free health services for children at all levels of the health system, as well as to provide free supplies of latrine construction materials.

#### Poor household economy

Discussions on the relative benefits of improved economic status as a basis for increased use of interventions and services did not take place: most of the participants were of similarly low economic status. The discussion regarding child feeding shows that the FGD participants’ knowledge of child feeding is high, indicating good knowledge brought about through the HEP, but participants openly admitted that most do not practice what they know, due to financial shortages to fulfil necessary foods. FGD participants explained the challenges related to child nutrition: “The first six months are not a problem as most of us feed only breast milk …. Otherwise we feed locally made bread ‘Injera’ and skimmed milk that we can get at home because majority of us couldn’t afford to prepare separate food.” One HEW also said that, “There are seven children under treatment for malnutrition in my health post, but many children are malnourished in this Woreda because of the poor feeding practices and a high prevalence of diarrhoea from poor sanitation practice.”

#### Low female autonomy

The FGD participants reflected that the use of household resources, participation in activities such as women’s development group meetings, and the need to seek maternal and child cereal require their husband’s permission. Low awareness of maternal and child health matters among the men worsen a woman’s ability to receive permission to undertake activities that promote family health. A HEW explained, “Husband dominance is high, and most women do not decide even over their time…. they need permission to participate in the Women’s Development Group, to receive maternal and child health services. If they do without permission, they may face quarrel and divorce.” This has implications for the implementation of health promoting interventions that they learn about from the HEWs.

### Theme: Behavioural factors

#### Seasonal mobility

The majority of the population in Wolkayit Woreda live in the highlands while their farming land is in the low-land. During the three- to four-month farming season, most able-bodied household members temporarily settle around their farming lands. At this time, it is a challenge for health workers to contact women for the regular WDG meetings and home visit programs, and the drop-out rates in maternal and child health care programs increase. Most of the health workers perceive this interrupted health promotion activity as being related to seasonal mobility, and as a major reason for poor environmental and reproductive health in the Woreda. One HEW said, “Home delivery increases and children do not come for treatment and services when adults, including women, move to the farming field for months. We reach mothers and children who remain at home only during [vaccination] campaigns.” A Primary health care directors indicated seasonal mobility as being a reason for the poor sanitation behaviour: “Knowing they are moving for more than four months a year; they do not want to build or do not give time to fix collapsed latrine.” A midwife added that, “Many children come with diarrhoea due to parasitic diseases caused by a shortage of potable water and poor sanitation behaviour. I have seen many people using the open field when they are asked to bring a stool sample even when they have latrines.”

#### Single motherhood

Single mothers often support themselves by selling local beverages and sticks for fuel, and they lack economic resources and support. FGD participants indicated that the social conditions brought about by this seasonal migration lead to many single motherhood, which in turn leads to poor child health status. The lack of a social support system and the need to make money leads to a shortage of time available to look after the children. One FGD participant reported that, “Afterwards, men consider that the child was born by the mother’s own choice and therefore many mothers raise their child as a single mother. We face a shortage of money and no time to prepare separate meals [for the young ones] since we spend the time selling sticks for fuel and carrying water.”

### Theme: Health system factors

The FGD participants explained that they trust in and accept the services at each level of the health system. Almost all of the FGD participants indicated Government-run Health Facilities as their preferred place of health care. Health Posts are accessible to and utilised by the majority of the community. However, a number of health system factors around access, availability and affordability were found to adversely affect uptake of child health care services at the higher level Health Centres and the Primary Hospital.

#### Accessibility to Health Centres and Hospitals

All FGD participants reported that the Health Post are sufficiently close to be within walking distance. This was confirmed by the health managers who reported that 93% of the villages have a Health Post nearby. The participants also stated that they trust the services they receive from health facilities to the extent that they do not use traditional healers to any great extent. However, major barriers exist to their accessing Health Centres and the Primary Hospital. Long distances for some people in remote areas, combined with a lack of transportation, poor roads, and rough topography make travelling to a health centre or hospital challenging, especially when carrying sick children or when in labour. One FGD participant stated that, “The Health Post is accessible to most of us, and we trust the services they provide us. We get many services including HIV testing for pregnant mothers. But we don’t get all the services that we need at the Health Post, and we are referred to the Health Facility that is more than eight hours walking from our village.” A HEW explained the challenge, saying, “Mothers do not want to be referred because of the long distance to the Health Centres. In my case, the nearest Health Centre that I refer to is eight hours walk from here. The community want to have all drugs in the Health Post but I am not trained to treat all diseases.” FGD participants suggested that road improvements and enhanced communication access for public and emergency ambulance transportation should be top priorities for the Government.

#### Unavailability of services

Due to supply stock outs and a lack of trained health workers, mothers with labour complications and sick neonates are often referred to the General Hospital, which is located over 60 km from the Woreda. Because of the inevitable delays brought about by this long distance, many arrive at the General Hospital with further complications and symptoms that are more difficult to manage than they would be had they arrived in good time. A clinician explained the problem, saying, “Most of the diseases are preventable and could have been treated at the Health Centre. But due to lack of emergency equipment for child and neonatal care service at the health post and the Health Centre, many referred mothers and children arrive with complications.”

As with the poor infrastructure in the Woreda reported by one study[27], we also observed that the majority of Health Posts lack electrical power. This problem impacts the vaccination campaigns and routine programs: it may lead to loss of vaccine potency due to a broken cold chain during transporting from the centre. Further, immunisation coverage for measles stands at 82% [24] which, according to the FGD participants, leads to regular measles outbreaks every year.

#### Unaffordable cost of services

Indirect costs, such as transportation, and fees for services provided at the health centre and Primary Hospital contribute to making health care unaffordable. Even though some services, such as skilled delivery, are offered free of charge to all, and additional service fees are waived for the poorest community members, many FGD and qualitative interview participants indicated that they purchase drugs, when they can afford them, from unlicensed sellers. One FGD participant explained the problem: “Even though we know it is better to go to the health centre, many of us do not afford 10 Birr (USD 0.50 cents) for the patient card only; or the additional charge for laboratory and drug. Therefore, we go to drug sellers who give us for less than 2 birr (USD 0.10 cents).” A Primary Health Care Unit director concurred: “Many people buy from unlicensed, ‘underground’ sellers where they can have doses which they can afford regardless of the doses needed, and which could be counterfeit. The women identify the drugs they used for similar illness by colour, and they use local names like ‘Segeni’ and ‘Amora’ for Ampicillin and Amoxicillin.”

## Discussion

Our study area is very remote and rural, and is characterized by a high prevalence of communicable diseases as well as the loss of many children from vaccine preventable diseases, malaria and diarrhoea. The FGD participants demonstrated good levels of knowledge about the major causes of morbidity and mortality of under-5 year children and of strategies that can be used to promote child health. It appears that the awareness-raising component of the HEP, which was designed with women’s and children’s health specifically in mind, has been working as intended [30]. This finding is consistent with studies that have shown that the HEP has been successful in improving maternal knowledge about under-5 year child health[31–32], particularly in communities with little or no formal education [12,33]. However, consistent with previous studies conducted in Ethiopia, our study found lower knowledge when it comes to the causes of neonatal morbidity and mortality, and this may well contribute to the slow reduction of neonatal mortality [34].

Although the health promotion and disease prevention knowledge was high, implementation of several cost-effective childhood services was not. We have observed that most of the maternal and child health interventions available at Health Posts—such as immunizations, bed nets to prevent malaria, antenatal care, and HIV testing—were all regularly accessed. Other services, however, such as interventions that require treatment for sick children from higher level facilities, improved child feeding practices, skilled delivery attendance, and neonatal care all had reportedly lower levels of access, even though knowledge about their availability was high. Poor utilization of these services by people in some communities inevitably leads to increased child health inequities. In order to address the cultural, financial and seasonal mobility barriers to health care utilization, a concerted, multi-sectoral effort is needed, including stakeholders from as the education, agriculture, justice, transportation, environment, and social security sectors.

We have described the different factors associated with the uptake of health care services according to Solar and Irwin’s (2010) conceptual framework of social determinants of childhood health inequities [25], which includes both structural and intermediary determinants. On this basis, we discuss our findings in the same four broad themes as they were presented in the Results.

### Cultural factors

We conducted our research in a culture that encourages home delivery, restricts mother’s movement after delivery, and hides the loss of babies. In this study, FGD participants did not want to contribute information regarding neonatal deaths, and some of them reportedly held positive attitudes about being restricted from moving in the weeks directly after delivery. Similar practices have been observed in other Ethiopian contexts [5,35] whereby, for example, a mother may be prevented from utilising health care from pregnancy all the way through the neonatal period. This cultural phenomenon contributes to higher risk of death both to the mother and the new-born, as it prevents early diagnosis and treatment from a health facility.

The lack of acknowledgement of the magnitude of under-5 child mortality by HEWs was unexpected. A possible explanation for this reported perception is the fact that they are responsible for preventing such deaths and for reporting those that do occur [31], and thus they may not want to admit to the real (higher) numbers. However, we also believe that they also are likely to be influenced by the culture in which they live, which tends to under-estimate child mortality. Further study is required on this. Hiding the loss of new-born babies by the community and by HEWs also encourages a false sense of security in the community concerning health outcomes, and it leads to the health system making decisions based on wrong information. This in turn could contribute to further promoting child health inequities in Tigray.

### Financial factors

Interventions that require financial or material expenses such as latrine development, uptake of child feeding practices, and treatment of sick children at Health Centres and higher facilities, were poorly utilized due to financial shortages to cover user fees as well as transportation and opportunity costs (e.g. lost wages). Globally, health status is most closely associated with income. Even where health care services are available, the cost of seeking care may delay or prevent poor households from accessing them and thereby increase the risk of both morbidity and mortality [33]. A recent study on the effectiveness of the HEP shows that when monetary expenses are required, the intervention was less effective, regardless of community knowledge of its potential benefits [36].

The low economic status and the high proportion of single mothers included in the study is likely correlated with the low reported usage rates of health services, as reported by the FGD participants. A lack of autonomy to make decisions regarding financial matters and when to seek health care, which leads to delayed or lack of health seeking, is one reason for poor uptake of child health interventions. In a culture where childcare is the responsibility of the mother and fathers are often away due to migrant farming patterns, this lack of autonomy further impacts negatively on health seeking behaviours.

Affordability of health care is a key determinant of child health equity [11,37]. A lack of affordable care leads to low or delayed utilization of services at the health centre and hospital. Low utilization contributes to inequities in health status, and can lead to self-prescribed treatment which may be ineffective at best or dangerous at worst. Even though a fee waiver and exemption system exists, the opportunity cost of seeking health care through lost wages makes potentially life-saving care unaffordable for a large proportion of the community.

### Behavioural factors

Every summer during the Ethiopian farming season, the majority of able-bodied household members in Wolkayit Woreda move from their homes, while additional people migrate into the Woreda from other regions to farm their own land or to work as daily labourers. During this period, they settle in temporary shelters which often lack access to adequate health facilities, clean water and sanitation. This relocation coupled with the inaccessibility of healthcare inhibits women and neonates from seeking basic preventive and curative care. Mobile communities are affected by water-borne diseases at higher rates than settled groups, and day labourers may reintroduce infectious diseases upon their return home. Both of these put children at higher risk of preventable conditions and may increase health inequities.

In addition, health promotion and Women’s Development Group activities are both interrupted during this migrant farming season [38]. Women face unique financial barriers and other gender-related barriers when a male head-of-household is away. These barriers prevent attendance at development group meetings; and they contribute to reduced health care-seeking behaviours. The high mobility of men also leads to an increase in out-of-marriage childbirth, which leaves women to raise children as a single mother with little or no financial or psychological support from the male partner. Single motherhood is correlated with higher child mortality[39]. The impact of seasonal mobility on population health, specifically child health, should be further studied.

### Health system factors

Distance to health care facilities and availability of transportation has been correlated with access to health care for children and with neonatal and under-five mortality [33,40]. Similarly in our study, long distance, mountainous topography, lack of road access, and poor telecommunication coverage in the Woreda [27] were all cited as barriers to accessing health care in a timely manner, or at all, by the majority of participants for treatment of sick child and delivery.

Several critical child health care services are available at the higher level health facilities, but not closer to the population through the Health Posts; this is true for treatment of pneumonia, diarrhoea, severe acute malnutrition, and new-born complications, all of which contribute significantly to under-5 mortality [40]. In addition, child health interventions such as prevention of diarrhoea and improved nutrition demand land, clean water, and money to buy materials. These cannot be addressed by the HEWs, as they are caused by larger, structural issues. The relative absence of these materials and assets in the Woreda plays an important role in increasing child health inequities in Tigray Region.

The shortage of HEWs to cover the wide geographical area over harsh topography was cited as the main reason for an inadequate numbers of home visits. Similarly, the low neonatal care knowledge of HEWs and the lack of neonatal care equipment in Health Centres is an additional barrier for access to child health services. This is consistent with findings elsewhere, whereby low efficiency [41] and low HEW knowledge of neonatal care [42] are contributing factors to the low uptake of skilled delivery and neonatal care, and consequently to the promotion of inequitable child health status.

## Conclusion

This study has generated important findings regarding the social determinants of child health in Wolkayit Woreda. The Ethiopian HEP is successful in terms of gaining the trust of the community and improving mothers’ knowledge about the causes and prevention of child mortality, except in the case of neonatal mortality. Consequently, most of the child health services and interventions that are provided at the health post level are well utilized. However, latrine construction and use, and child feeding interventions are poorly practiced despite high levels of knowledge. Child health services provided at health centre and hospital level such as delivery and treatment of sick children are generally poorly utilized.

Intertwined determinants such as cultural and traditional factors, financial factors, seasonal mobility, and health system factors were identified as contributing to the poor implementation of child health services. Cultural barriers were seen as responsible for the poor utilization of maternal and neonatal health care. Household financial factors complemented with inaccessible health facilities resulted in poor implementation of interventions and decreased health-seeking behaviours for maternal and child health. Lastly, seasonal mobility was identified as an important barrier to accessing health promotion and health care services.

## Supporting information

S1 Table Guiding questions for FGD and qualitative interview in local language, Tigrigna. (DOCX)

S2 Table Guiding questions for FGD and qualitative interview in English. (DOCX)
