# Supplementary material for: Social determinants of under-5 child health: A qualitative study in Wolkayit Woreda, Tigray Region, Ethiopia
Source: PLoS One. 2019 Jun 13;14(6):e0218101. doi: 10.1371/journal.pone.0218101 (PMC6564425; doi:10.1371/journal.pone.0218101)
Supplement: S1 Table — (DOCX) [file pone.0218101.s001.docx]

S1 Table. መራሒ መሕቶት ንጥሙት ሓበራዊ ምይይጥን ሓደ ንሓደ ሕቶን መልስን ምስ በዓልሞያታት ጥዕና፡፡

መፅናዕቲ ማህበራዊ ምኽንያታት ኩነታት ጥዕና ትሕቲ 5 ኣመት ቆልዑ አብ ወለወቃይት ወረዳ፡ ትግራይ ፡ ኢትዮጵያ፡፡ ምይይጥ መሰረት ዝገበረ መፅናዕቲ፡ አታኽለቲን መሳርሕቱን

| **ተ.ቁ** | **መሕቶት ንጥሙት ሓበራዊ ምይይጥ (አዴታት)** | **ትኹረት ሕቶታት** |
| --- | --- | --- |
|  | ትሕቲ 5- ዓመት ቆልዓ አለኪዶ? | - ብሂዎት አሎዶ? - ኩነታት ጥዕንኡ ከመይ ትገልፅዮ? |
|  | ኩነታት መነባበሮኸን ከመይ ትርእየኦ? | - ምኽንያቱ እንታይ እዩገ - ካብህሉፍ ለወጢ አለዎዶ? |
|  | ኩነታት ጥዕና አብዚ ከባቢ ዘለው ቆልዑ ከመይ ትግምግመ? | - ምስ ካልኦት ወረዳታት ትግራይ እንትነፃፀር ? - ካብ ሕሉፍ እንትነፃፀር ? - ንምንታይ? |
|  | አብ ሎሚ እዋን ቀንዲ ምክንያታት ፀገም ጥዕና ቆልዑ እንታይ እዮም? | - ካሊእከ? |
|  | ቆልዓ ንምውላድ እትመርፂዮ ቦታ/ትካል አበይ እዩ ? | - እንታይ ምኽንያት አለኪ? - አብቲ ቦታ እንታይ ዓይነት ግልጋሎታት ትረኽቢ? |
|  | ገልጋሎት ኤች አይ ቪ አበይ ትረኽቢ? | - እንታይ ዓነት ግልጋሎት ትረኽቢ? - እቲ ግልጋሎት ከመይ ትሪኢዮ? |
|  | አብ ከባቢኹም ዓሶ ፀገም ጥዕና ቆልዑ ድዩ? | - ዓሶ ብከመይ ትከላኸልዮን ትሕከምዮን?አጎበር ትጥቀሚዶ? - ቆልዓ ዓሶ እንተሓምም እነታይ ስጉምቲ ትወስዲ? ንምነታይ? |
|  | አብ ከባቢኹም ብቅፅበታዊ ስርዓት ምስትንፋስ ፀገም ጥዕና ቆልዑ ድዩ? | - ቆልዓኪ ብቅፅበታዊ ስርዓት ምስትንፋስ እንተሓምም ትፈልጢዶ? - ካብ ቀሊል ሰዓል ብኸመይ ትፈልይዮ - እንተጋጥሞ እንታይ ስጉምቲ ትወስዲ? |
|  | አብ ከባቢኹም ውፅአት ፀገም ጥዕና ቆልዑ ድዩ? | - ውፅአት ብከመይ ትከላኸልዮን ትሕከምዮን? - ቆልዓ እንተሓምም እነታይ ስጉምቲ ትወስዲ? ንምንታይ? |
|  | አመጋግባ ትሕቲ 5- ዓመት ቆልዓ ትፈልጢዶ? | - አብከባቢኺ አዴታት ንደቀን ከመይ ይምግባ ? - ምኽንያቱ እንታይ እዩ? - ንቆልዑ ፍሉይ መግቢዶ ይዳለው ወይስ ምስዓበይቲ እዮም ዝምገቡ? |
|  | አቦ ቆልዓ አብ ምክንኻን ደቆም ዘለዎም ግደ እንታይ ይመስል? | - እንታይ ግደ ክህልዎ ይግባእ? - ምኽንያቱ እንታይ እዩ? |
|  | ንመራሕቲ ዘፈር ጥዕና አብ ወልቃይት ዘሎ አወሃህባ ግልጋሎት ጥዕና አመልኪቱ ተዝከውን እትብልዮ ሓሳብ እንታይ እዩ? | - ክቅፅል ዝግበኦ? - ክመሓየሽ ዝግበኦ? - ካሊእ ትብለኦ አሎ? |
|  | **መሕቶት ንሓካይም** |  |
| 1 | አብዚ ሆሰፒታል ክንደይ ዓመት ሰሪሕካ/ኪ ? | - ዓመት…….. ወርሒ……..? - አብስራሕኪ/ካ ሕጉስ ዲኻ/ኺ? - ንምንታይ? |
| 2 | አብ ፃንሒትካ/ኪ ከምትዕዘቦ ናብዚ ሆሰፒታል ሪፈር ተባሂሎም ካብዝመፁ ቆልዑ ዋና ዋና ምኽንያታት ሕማምን ሞትን ትሕቲ 5 ዓመት ቆልዑ እንታይ እዩ ? | - ብቅደም ሰዓብ - አብሕንጠያትከ? - ካሊእከ? |
| 3 | ክንደይ ናይ ኤችአይቪ ሕክምና ዝወስዱ ትሕቲ 5 ዓመት ቆልዑ አለዉ ? | - ሕክምነኦም ብአግባቡ ንምውሳዶም ከመይ ትከታተሎም? |
| 4 | ብሕማም ሳምቡእ/ ሳምባ ምቺ፤ ሕማም ውፅአት፤ ሕማም ዓሶ ናብዚ ሆስፒታል ዝመፁ ቆልዑ አብ ጣብያ ጥዕናን ክሕከሙ ዝኽእሉ እዮም ኢልካ/ኪ ዶ ትሓስቢ? | - ምኽንያቱ እነታይ እዩ ይመፁ? - ምፍትሒኡ እንታይ እዩ ትብል/ሊ? |
| 5 | ካብ ወረዳ ወልቃይት ናብዚ ሆሰፒታል ዝመፁ ቆልዑ ኩሎም ወረቀት ሪፈራል ሒዞም ዝመፁ ድዮም ወይስ ብድልየቶም እዮም ዝመፁ? | - ንምንታይ? |
| 6 | ትሕቲ 5 ዓመት ቆልዑ ብሪፈራል ይኹን ብባዕሎም ናብዚ ሆሰፒታል እንተመፁ ኩነታቶም እንታይ ይመስል? | - ሕማም እንተይገደዶም ዶ ይመፁ? ወይስ ብጣዕሚ ገዲድዎም ? - ምኽንያቱ እንታይ እ ዩ ትብል/ሊ |
| 7 | ምጣነ ሞት ትሕቲ 5 ዓመት ቆልዑ ወልቃይት ምስ ልዕሊ ማእኸላይ መጠን ሞት ትግራይ እዩ ? | - ኢልካ ትአምንዶ? - ምኽንያቱ እንታይ እዩ ትብል? |
| 8 | ንመራሕቲ ዘፈር ጥዕና አብ ወልቃይት ከባቢኪ ስለዘሎ አወሃህባ ግልጋሎት ጥዕና ተዝከውን እትብልዮ ሓሳብ እንታይ እዩ? | - ክቅፅል ዝግበኦ? - ክመሓየሽ ዝግበኦ? - ካሊእ ትብለኦ አሎ? |
|  | **መሕቶት ንነርስታት** |  |
| 1 | አብዚ ጣብያ ጥዕና /ሆሰፒታል ክንደይ ዓመት ሰሪሕካ/ኪ ? | - ዓመት…….. ወርሒ……..? - አብስራሕኪ/ካ ሕጉስ ዲኻ/ኺ? - ንምንታይ? |
| 2 | አብ ፃንሒትካ/ኪ ዋና ዋና ምኽንያታት ሕማምን ሞትን ትሕቲ 5 ዓመት ቆልዑ እንታይ እዩ ? | - ብቅደም ሰዓብ - አብሕንጠያትከ? - ካሊእከ? |
| 3 | ንሕብረተሰብ ብዛዕባ ሕማም ስርዓት ምስትንፋሰ አስተምህሮ ጥዕና ትህቢ/ብ ዶ? | - ሕብረተሰብ ንሕማም ስርዓት ምስትንፋሰ ቆልዑ ብኸመይ ይርድኦ ? - ህብረተሰብ ቀሊል ሰዓልን ቅፅበታዊ ሕማም ስርዓት ምስተንፋስን ፈልዩ ይፈልጥ ዶ ትበሊ/ል? |
| 4 | አብ ከባቢኺ መጠን ዝርገሐ ሕማም ውፅአት አብ ቆልዑ ከመይ ትገልፅዮ/ፆ? | - ምኽንያቱ እንታይ እዩ? - ሕብረተሰብ ከመይ የከላኸሎን ይሕከሞን? |
| 5 | አብ ከባቢኺ መጠን ዝርገሐ ሕማም ዓሶ አብ ቆልዑ ከመይ ትገልፅዮ/ፆ? | - ምኽንያቱ እንታይ እዩ? - ሕብረተሰብ ንሕማም ዓሶ ከመይ የከላኸሎን ይሕከሞን? |
| 6 | አብዚ ወረዳ ቆልዑ እንትሓሙ ከመይ ይሕክምዎ? | - መጀመርታ ናበይ ይወስድዎም? - ንምንታይ? |
| 7 | ወለዲ ንዝሃመሙ ቆለዑ ናብ ሕክምና ቀልጢፎም የምፅኡዶ ? | - ከይተዳኸሙ? ብጣዕሚ ገዲድዎም? - ምኽንያቱ እንታይ እ ዩ ትብል/ሊ? |
| 8 | ሕብረተሰብ ብበዓል ሞታት ዝውሃበ ምኽሪን ሕክምናን ቆልዓ ተቐቢሎም ይትግበሩዶ? | - ብኸመይ ጥፈልጦ/ጥዮ? - ንምንታይ? |
| 9 | አብዚ ትካል ግልጋሎት ሕክምና ፀረ ኤች አይ ቪ ትህቡዶ? | - ድገፍን ክትትልን ከመይ ይፍፀም ? |
| 10 | ሞት ትሕቲ 5 ዓመት ቆልዑ ወልቃይት ወረዳ ልዕሊ ማእኸላይ መጣነ ሞት ክልል ትግራይ እዩ ? | - ኢልካ ትአምንዶ? - ምኽንያቱ እንታይ እዩ ትብል? |
| 11 | ንመራሕቲ ዘፈር ጥዕና አብ ወልቃይት ከባቢኪ ስለዘሎ አወሃህባ ግልጋሎት ጥዕና ተዝከውን እትብልዮ ሓሳብ እንታይ እዩ? | - ክቅፅል ዝግበኦ? - ክመሓየሽ ዝግበኦ? - ካሊእ ትብለኦ አሎ? |
|  | **መሕቶት ሚደዋይፍ ነርስታት** |  |
| 1 | አብዚ ጣብያ ጥዕና/ ሆሰፒታል ክንደይ ዓመት ሰሪሕካ/ኪ ? | - ዓመት…….. ወርሒ……..? - አብስራሕኪ/ካ ሕጉስ ዲኻ/ኺ? - ንምንታይ? |
| 2 | አዴታት ወልቃይት እንትወልዳ አበይ ይመርፃ ? | - ጣብያ ጥዕና? ሆስፒታል? ገዛ? - አብገዛ እንተወልዳ ምን የዋልደን? |
| 3 | ካብ ተሞክሮኺ አብዚ ኸባቢ መጠን ሞት ሕንጠያት ከመይ ጥገልፅ? | - ንምንታይ? - ምኽንያት ሞት እንታይ እዩ ትብሊ? |
| 4 | ንሕብረተሰብ ብዛዕባ ናይ ሕንጠያት ቅፅበታዊ ሕማም ስርዓት ምስትንፋሰ አስተምህሮ ጥዕና ትህቢ/ብ ዶ? | - ሕብረተሰብ ብኸመይ ይርድኦ ? |
| 5 | አብ ከባቢኺ መጠን ዝርገሐ ሕማም ውፅአት አብ ቆልዑ ከመይ ትገልፅዮ/ፆ? | - ምኽንያቱ እንታይ እዩ? - ሕብረተሰብከመይ የከላኸሎን ይሕከሞን? |
| 6 | አብ ከባቢኺ መጠን ዝርገሐ ሕማም ዓሶ አብ ቆልዑ ከመይ ትገልፅዮ/ፆ? | - ምኽንያቱ እንታይ እዩ? ሕብረተሰብ ንሕማም - ዓሶ ከመይ የከላኸሎን ይሕከሞን? |
| 7 | አብዚ ወረዳ ሕንጠያት እንትሓሙ ከመይ ይሕክምዎ? | - መጀመርታ ናበይ ይወስድዎም? - አብገዛ እንታይ ይግበረሎም? |
| 8 | ወለዲ ንዝሃመሙ ሕንጠያት ናብ ሕክምና ቀልጢፎም የምፅኡዶ ? | - ከይተዳኸሙ? - ብጣዕሚ ገዲድዎም ? - ምኽንያቱ እንታይ እ ዩ ትብል/ሊ? |
| 9 | አብዚ ትካል ግልጋሎት ሕክምና ፀረ ኤች አይ ቪ ትህቡዶ? | - ድገፍን ክትትልነ ቆልኡ ከመይ ይፍፀም ? |
| 10 | ምጣነ ሞት ሕንጠያት ወልቃይት ልዕሊ ማእኸላይ ክልል ትግራይ እዩ ? | - ኢልካ ትአምንዶ? - ምኽንያቱ እንታይ እዩ ትብል? |
| 11 | ንመራሕቲ ዘፈር ጥዕና አብ ወልቃይት ከባቢኪ ስለዘሎ አወሃህባ ግልጋሎት ጥዕና ተዝከውን እትብልዮ ሓሳብ እንታይ እዩ? | - ክቅፅል ዝግበኦ? - ክመሓየሽ ዝግበኦ? - ካሊእ ትብለኦ አሎ? |
|  | **መሕቶት ጥሙር ጥዕና ቤተሰብ** |  |
| 1 | አብዚ ኬላ ጥዕና ክንደይ ዓመት ሰሪሕኪ ? | - ዓመት…….. ወርሒ……..? - አብስራሕኪ ሕጉስቲ ዲኺ? - ንምንታይ? |
| 2 | ብዛዕባ ዘለኪ ስራሕን ሓላፍነትን እንታይ እዩ? | - ብኸመይ? - ገዛገንገዛ ብምኻድ? - ካሊእ? |
| 3 | ገዛ ንገዛ እንዳኸድኪ እትህብዮ ግልጋሎት እንታይ እዩ? | - በቢክንደይ እዋን ተጉብንዪ? እንታይ ስራህቲ ትሰርሒ? |
| 4 | አብ ኬላ ጥዕና እትህብዮም ግልጋሎት ሕክምና እንታይ እዮም ? | - ዘድሊ እኹል ናውቲ ሕክምናን አፋውስን አለኪዶ? |
| 5 | ሕብረተሰብ አብቲ እትህብ ግልጋሎት ተቀባልነትን እምንተ አለዎዶ ? | - ብኸመይ ይገልፀልኩም? |
| 6 | ካብ ተሞክሮኺ ምኽንያታት ሕማምን ሞትን ትሕቲ 5 ዓመት ቖልዑ እዚ ኸባቢ እንታይ እዩ ኢልኪ ትሓስቢ? | - ብቅደም ሰዓብ - አብሕንጠያትከ? - ካሊእከ? |
| 7 | አብ ከባቢኺ መጠን ዝርገሐ ሕማም ውፅአት አብ ቆልዑ ከመይ ትገልፅዮ/ፆ? | - ምኽንያቱ እንታይ እዩ? - ሕብረተሰብ ከመይ የከላኸሎን ይሕከሞን? |
| 8 | አብ ከባቢኺ መጠን ዝርገሐ ሕማም ዓሶ አብ ቆልዑ ከመይ ትገልፅዮ/ፆ? ምኽንያቱ እንታይ እዩ? ሕብረተሰብ ንሕማም ዓሶ ከመይ የከላኸሎን ይሕከሞን? | - ምኽንያቱ እንታይ እዩ? - ሕብረተሰብ ከመይ የከላኸሎን ይሕከሞን? |
| 9 | ንሕብረተሰብ ብዛዕባ ናይ ሕንጠያት ቅፅበታዊ ሕማም ስርዓት ምስትንፋሰ አስተምህሮ ጥዕና ትህቢ/ብ ዶ? | - ሕብረተሰብ ብኸመይ ይርድኦ ? - እንታይ መማረፂ ይወስድ ? |
| 10 | አዴታት ወልቃይት እንትወልዳ አበይ ይመርፃ ? | - ጣብያ ጥዕናዶ? አብገዛ? - አብገዛ እንተወልዳ መን የዋልደን? |
| 11 | ኬላ ጥዕና ንህዝቢ ጣብያ ተበፃሓይ እዩዶ ትብሊ? | - ብኸመይ ትፈልጢ? |
| 12 | አብዚ ወረዳ ቆልዑ እንትሓሙ መጀመርታ እንታይ ስጉምቲ ይውሰድ? | - መጀመርታ ናበይ ይወስድዎም? - አብ ገዛ ከመይ ይሕክምዎ? |
| 13 | እትህበዮም ምኽሪን ተቐቢሎም ይትግበሩዶ? | - እንታይ ብፅቡቅ ይቅበሉ? - አበየናይ የፅግም? |
| 14 | ሞት ትሕቲ 5 ዓመት ቆልዑ ወልቃይት ልዕሊ ማእኸላይ ምጣነ ትግራይ እዩ ? | - ኢልካ ትአምንዶ? - ምኽንያቱ እንታይ እዩ ትብሊ? |
| 15 | ንመራሕቲ ዘፈር ጥዕና አብ ወልቃይት ከባቢኪ ስለዘሎ አወሃህባ ግልጋሎት ጥዕና ተዝከውን እትብልዮ ሓሳብ እንታይ እዩ? | - ክቅፅል ዝግበኦ? - ክመሓየሽ ዝግበኦ? - ካሊእ ትብለኦ አሎ? |
|  | **መሕቶት አመራርሓን ትካላት ጥዕና** |  |
| 1 | አብዚ ወረዳ ብሓላፍነትን ብሞያን ክንደይ ዓመት ሰሪሕካ/ኪ ? | - ዓመት…….. ወርሒ……..? - ስራሕን ሓላፍነትኪ/ካ እንታይ እዩ? - አብስራሕኪ/ካ ሕጉስ ዲኻ/ኺ? - ንምንታይ? |
| 2 | ዋና ዋና ምኽንያታት ሕማምን ሞትን ትሕቲ 5 ዓመት ቆልዑ ወልቃይት እንታይ እዩ ትብል/ሊ? | - ብቅደም ሰዓብ - አብሕንጠያትከ? - ካሊእከ? |
| 3 | ንሕብረተሰብ ብዛዕባ ሕማም ስርዓት ምስትንፋሰ አስተምህሮ ጥዕና ትህቢ/ብ ዶ? | - ብኸመይ ይርድኦ ? - ቀሊል ሰዓልን ቅፅበታዊ ሕማም ስርዓት ምስተንፋስን ፈልዩ ይፈልጥ ዶ ትበሊ/ል? |
| 4 | አብ ከባቢኺ መጠን ዝርገሐ ሕማም ውፅአት አብ ቆልዑ ከመይ ትገልፅዮ/ፆ? | - ምኽንያቱ እንታይ እዩ? - ሕብረተሰብ ከመይ የከላኸሎን ይሕከሞን? |
| 5 | አብ ከባቢኺ መጠን ዝርገሐ ሕማም ዓሶ አብ ቆልዑ ከመይ ትገልፅዮ/ፆ? | - ምኽንያቱ እንታይ እዩ? - ሕብረተሰብ ከመይ የከላኸሎን ይሕከሞን? |
| 6 | አብዚ ወረዳ ቆልዑ እንትሓሙ መጀመርታ እንታይ ስጉምቲ ይውሰድ? | - መጀመርታ ናበይ ይወስድዎም? - አብ ገዛ ከመይ ይሕክምዎ? |
| 7 | ወለዲ ንዝሃመሙ ቆለዑ ናብ ሕክምና ቀልጢፎም የምፅኡዶ ? | - ከይተዳኸሙ? - ገዲድዎም ? - ምኽንያቱ እንታይ እ ዩ ትብል/ሊ? |
| 8 | ብበዓል ሞያታት ዝተውሃበ ምኽሪን አስተምህሮን ተቐቢሎም ይትግበሩዶ? ን | - ንምንታይ? - ብኸመይ ይፍለጥ? |
| 9 | ሕክምና ፀረ ኤች አይ ቪ አበይ አበይ ይወሃብ? | - ስርዓት ክትትል ከመይ ይፍፀም ? |
| 10 | ሞት ትሕቲ 5 ዓመት ቆልዑ ወልቃይት ልዕሊ ማእኸላይ ምጣነ ሞት ትግራይ እዩ ? | - ኢልካ ትአምንዶ? - ምኽንያቱ እንታይ እዩ ትብል? |
| 11 | ንመራሕቲ ዘፈር ጥዕና አብ ወልቃይት ከባቢኪ ስለዘሎ አወሃህባ ግልጋሎት ጥዕና ተዝከውን እትብልዮ ሓሳብ እንታይ እዩ? | - ክቅፅል ዝግበኦ? - ክመሓየሽ ዝግበኦ? - ካሊእ ትብለኦ አሎ? |

የቐንየለይ፡፡ ሕቶታትና ወዲእና አለና ኾየኑ ግን ካሊእ ዘይተገለፀ አድላዪ ጉዳይ እንተልዩ ክቕበለኩም ድልውየ፡፡
