# Supplementary material for: Social determinants of under-5 child health: A qualitative study in Wolkayit Woreda, Tigray Region, Ethiopia
Source: PLoS One. 2019 Jun 13;14(6):e0218101. doi: 10.1371/journal.pone.0218101 (PMC6564425; doi:10.1371/journal.pone.0218101)
Supplement: S2 Table — (DOCX) [file pone.0218101.s002.docx]

S2 Table. Guiding questions for the Focus Group Discussions and for the qualitative Interviews on the social determinants of under-5year child health status in Wolkayit Woreda, Tigray Region, Ethiopia: a qualitative study – Abraha et al

| **S.No** | **Guiding Questions for the Focus Group Discussions (mothers)** | **Probing questions** |
| --- | --- | --- |
|  | Do you all have children under-5 year children? | - Are all alive today? - How do you see their health condition? |
|  | How do you see your and the communities living condition? | - Why is that? - How do you compare with past years? |
|  | How do you evaluate the current health status of children | - compared to how things were when you were a child? - compared to other districts in Tigray? And why is that? |
|  | What are the major causes of child illness? | - What else? |
|  | Where do you think is a good place to deliver a baby? | - Why is that? Is distance a reason? - What services do you get there? - what do you think about these services? What services do you |
|  | Where do people here receive HIV related services? | - What services do you get? - How do you see the services that you get there? |
|  | Tell me about Malaria as a child health problem in this area? | - What do you do to prevent and treat malaria? what about having and using bed nets? - Where and how often do you go for treatment of malaria? Why? |
|  | Tell me about acute respiratory tract infections as a child health problem in this area? | - What do you do to prevent and treat ARI? - How do you differentiate from flue or common cold? - What do you do when children get ARI? |
|  | Tell me about diarrhea as a child health problem in this area? | - What do you do to prevent and treat diarrhea? - What do you do when children get diarrhea? |
|  | Are you aware of ideal feeding for under-5 year children? | - What do you think is the ideal? - Is this available here? - How do mothers in this community manage to feed their children? - Do children eat together with adults or there is there some other sort of arrangement? |
|  | How do you see the role of your children’s’ father in taking care of the children? | - What roles do you think they should have? - Why is that? |
|  | What recommendations do you have for the people who make decisions about the health services here in Wolkayit? | - Good need to continue? - Need improvement? - Other? |
|  | **Qualitative interview questions for Clinicians** |  |
|  | How long have you worked in this hospital? | - Year------------- months--------- - Are you happy being here? - Why? |
|  | In your experience what do you see as the main causes of morbidity and mortality for under-5 year children coming from Wolkayit Woreda? | - The top - In neonates? Infants? - What else? |
|  | How many children do you have in your care receiving anti-retroviral therapy (for HIV infection)? | - How do you follow and support children receiving ART to make sure they are taking their drugs as per the prescription you ordered? |
|  | Do you think diseases such as pneumonia, diarrhea and malaria could have been treated in their nearest health center? | - Why are they coming to the Hospital? - How could this situation be improved? |
|  | Do you think ill children from the Woreda come to hospital by referral from the nearest health institution, or are they self-referred? | - Why is that? |
|  | Do you think under-5 year children are coming for treatment in good time? | - How do you consider the severity of illness of the children to be upon arrival? And why is that? |
|  | The mortality of under-5 year children in this Woreda is higher than the regional average, | - Do you think so? - Why is that? |
|  | What recommendations do you have for the people who make decisions about the health services here in Wolkayit? | - Good need to continue? - Need improvement? - Other? |
|  | **Qualitative interview questions for Nurses** |  |
|  | How long have you worked in this hospital/Health center? | - Year------------- months--------- - Are you happy being here? - Why? |
|  | In your experience what do you see the causes of morbidity and mortality under-5 year children coming from your catchment area? | - The top - In neonates? Infants? - What else? |
|  | To what extent do you see ARIs as a problem in the community? | - Do you educate the community about ARIs? - How the communities understand - Do they differentiate from simple cough and URTI? - What do they do for ARI? At home? |
|  | To what extent do you see diarrheal disease as a problem in the community? | - Why is that? - How do the community prevent diarrhea? Treat diarrhea? |
|  | To what extent do you see malaria as a problem in the community? | - How do the community here prevent and treat malaria? |
|  | Where are children taken first if they are sick? | - Why? |
|  | How soon and how often do parents bring their children for treatment when they get sick? | - come in good time? - How do you consider the severity of illness of the children to be upon arrival? - Why is that? |
|  | Do you trust parents to follow the instructions and advice given by you regarding medication and related issues? | - Why/why not? |
|  | Do you provide ART for children here? | - How do you follow and support children under ART to make sure they are taking their drugs as per the prescription? |
|  | The mortality of under-5 year children in this Woreda is higher than the regional average? | - Do you agree? - why is that? |
|  | What recommendations do you have for the people who make decisions about the health services here in Wolkayit? | - Good need to continue? - Need improvement? - Other? |
|  | **Qualitative interview questions for Midwives** |  |
|  | How long have you worked in this hospital/Health center? | - Year------------- months--------- - Are you happy being here? - Why? |
|  | Do most mothers in your community deliver at the health center? | - What are the reasons? - Who is attending the labor of the remaining mothers? |
|  | How do you see the status of neonatal mortality in your area? | - Why is that? - What do you think the major causes are? |
|  | How do you educate the community about neonatal pneumonia? | - How well do you think the communities understand this issue? |
|  | How do you educate the community about neonatal sepsis? | - How well do you think the communities understand this issue? |
|  | To what extent do you see diarrheal disease as a problem in the community? And why is that? | - Why is that? - How do the community prevent and treat diarrhea? |
|  | To what extent do you see malaria as a problem in the community? | - How do the community here prevent and treat malaria? |
|  | Where are neonate children first taken if they are sick? Why? | - Why is that? - What actions they take at home? |
|  | Do parents bring their neonate child for treatment when they get sick? | - How soon and how often? - Come in good time? - How do you consider the severity of illness upon arrival? - Why is that? |
|  | Do you trust parents to follow the instruction and advice given by you regarding medication and related issues? | - Why/why not? |
|  | The mortality of neonates in this Woreda is higher than the regional average, | - Do you agree? - Why is that? |
|  | What recommendations do you have for the people who make decisions about the health services here in Wolkayit? | - Good need to continue? - Need improvement? - Other? |
|  | **Qualitative interview questions for Health Extension Workers** |  |
|  | How long have you worked in this Health Post? | - Year------------- months--------- - Are you happy being here? - Why? |
|  | Can you tell me your duties and responsibilities? | - What else? - Do you think you understand the child health problems in this kebele? |
|  | Do you visit households for your work? | - How often? - What do you do when you go to the households? |
|  | What illnesses do you treat in the health post? | - Is that adequate for the community? What other services do they ask? - Do you have adequate facilities to treat these? |
|  | Do the community trust and accept your treatment and health advice? | - What is the feedback from the community ? |
|  | In your experience what do you see the causes of morbidity and mortality under 5 year children in your area? | - The top - In neonates? Infants? - What else? |
|  | To what extent do you see diarrheal disease as a problem in the community? And why is that? | - Why is that? - How do the community prevent diarrhea? Treat diarrhea? |
|  | To what extent do you see malaria as a problem in the community? How do the community here prevent and treat malaria? | - How do the community here prevent and treat malaria? At home? |
|  | How do you educate the community about ARIs? How the communities understand and differentiate between simple cough and URTI? | - Do you educate the community about ARIs? - How the communities understand - Do they differentiate from simple cough and URTI? - What do they do for ARI? At home? |
|  | Where are children taken first if they are sick? Why? | - Why? |
|  | Do you think health post is accessible to the community? | - Why/why not? |
|  | How soon and how often do parents bring their child for treatment when they get sick? | - Come in good time? - How do you consider the severity of illness of the children to be upon arrival? |
|  | Do you trust parents to follow the instruction and advice given by you regarding medication and related issues? | - Why/why not? |
|  | The mortality of children in this Woreda is higher than the regional average? | - Do you agree? - why is that? |
|  | What recommendations do you have for the people who make decisions about the health services here in Wolkayit? | - Good need to continue? - Need improvement? - Other? |
|  | **Qualitative interview questions for health managers** |  |
|  | How long have you worked in this position in the Woreda? | - Year------------- months--------- - Are you happy being here? - Why? |
|  | Can you tell me your duties and responsibilities? | - What else? - Do you think you understand the child health problems in this district? |
|  | In your experience what do you see as the main causes of morbidity and mortality for under-5 year children in Wolkayit Woreda? | - The top - In neonates? Infants? - What else? |
|  | Is ARI cause of morbidity and mortality in your district? | - Why is that? - How do you educate the community about ARIs? - How the communities understand and differentiate between simple cough and URTI? |
|  | To what extent do you see diarrheal disease as a problem in the community? | - Why is that? - How do the community prevent and treat diarrhea? |
|  | To what extent do you see malaria as a problem in the community? | - How do the community here prevent and treat malaria? |
|  | Where do parents take their children first if they are sick? Why? | - Why is that? - What actions they take at home? |
|  | How soon and how often do parents bring their child for treatment when they get sick? | - Coming in good time? - How do you consider the severity of illness of the children to be upon arrival? - Why is that? |
|  | Do you trust parents to follow the instruction and advice given by you regarding medication and related issues? | - Why/why not? |
|  | How do see the HIV services in the district? | - What services do you provide? - How do you follow and support children receiving ART to make sure they are taking their drugs as per the prescription? |
|  | The mortality of under-5 year children in this Woreda is higher than the regional average, why is that? | - Do you agree? - Why is that? |
|  | What recommendations do you have for the people who make decisions about the health services here in Wolkayit? | - Good need to continue? - Need improvement? - Other? |

Thank you for your time and cooperation.

I am ready to hear if you have any think that you think important but not discussed.
